# Five year outcomes of primary and secondary Single-Anastomosis Duodeno-Ileal bypass with Sleeve gastrectomy (SADI-S)

**DOI:** 10.1007/s11695-025-07888-4

**Published:** 2025-05-09

**Authors:** Mitchell J. R. Harker, Laura Heusschen, Valerie M. Monpellier, Ronald S.L. Liem, Magaly J.J. Van himbeeck, Simon W. Nienhuijs, May Al Nawas, Rene J. Wiezer, Guusje Vugts, Eric J. Hazebroek

**Affiliations:** 1 Department of Bariatric Surgery, Vitalys, part of Rijnstate Hospital, Arnhem, Netherlands; 2 Division of Human Nutrition and Health, Wageningen University, Wageningen, Netherlands; 3 Dutch Obesity Clinic, Huis ter Heide, Netherlands; 4 Department of Surgery, Catharina Hospital Eindhoven, Eindhoven, Netherlands; 5 Department of Surgery, St. Antonius Hospital, Nieuwegein, Netherlands

**Keywords:** Sleeve gastrectomy, Single-anastomosis duodeno-ileal bypass, SADI-S, Conversion surgery, Secondary surgery, Recurrent weight gain

## Abstract

**Background:**

The single-anastomosis duodeno-ileal bypass with sleeve gastrectomy (SADI-S) can be performed as a primary or (planned) secondary metabolic bariatric procedure. The aims of this study were to compare mid-term outcomes up to 5 years after primary vs secondary SADI-S and between different common channel (CC) lengths.

**Methods:**

Multicenter retrospective cohort study including 103 patients who underwent SADI-S between 06–2015 and 02–2019. Outcomes on weight loss, nutrient status, health-related quality of life (HRQoL) and gastro-intestinal symptoms until 5 years postoperatively were evaluated and compared between primary (*n* = 19) vs secondary SADI-S (*n* = 84), and CC length ≤ 250 cm (*n* = 66,) vs > 250 cm (*n* = 33).

**Results:**

Mean total weight loss (TWL) at 5 years of follow-up was higher for patients who underwent primary SADI-S compared to secondary SADI-S (34.8 (29.8–39.9)% vs 15.9 (13.0–18.9)%, *p* < 0.001) and for CC length ≤ 250 cm compared to > 250 cm (25.3 (21.8–28.9)% vs 21.3 (17.2–25.4)%, *p* = 0.12). Patients who underwent primary SADI-S also had significantly higher scores on the domains of the BODY-Q HRQoL questionnaire (*p* < 0.05 for all), with the exception of sexual well-being. Nutrient status and gastro-intestinal symptoms were comparable between the indication groups, but CC length ≤ 250 cm tended to result in more nutrient deficiencies and higher defecation frequency.

**Conclusion:**

Both primary and secondary SADI-S result in durable weight loss outcomes up to 5 years postoperatively. It is imperative that CC length should be at least 250 cm to prevent malnutrition and gastro-intestinal complaints. Furthermore, focus on HRQoL is essential in future research into SADI-S.

**Graphical Abstract:**

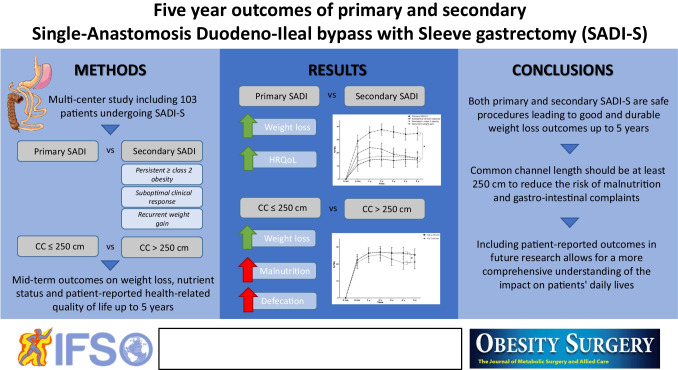

**Supplementary Information:**

The online version contains supplementary material available at 10.1007/s11695-025-07888-4.

## Introduction

Sleeve gastrectomy is the most performed metabolic bariatric surgical procedure worldwide [1], and can be performed either as a stand-alone or as a first stage procedure in patients with a body mass index (BMI) ≥ 50 kg/m^2^ [2]. Multiple surgical techniques are available as conversion procedure or planned second step after SG, including single-anastomosis duodeno-ileal bypass (SADI), Roux-en-Y gastric bypass (RYGB), one anastomosis gastric bypass (OAGB) and duodenal switch (DS) [3–5].

In 2007, Sánchez-Pernaute et al. first introduced the SADI with SG (SADI-S) as a single step procedure [6], with total weight loss (TWL) ranging between 22.5–38.0% beyond five years postoperatively [7–9]. Later, the effectiveness of the SADI as a second step after SG was also shown with TWL ranging between 15.0–41.0% beyond five years postoperatively [7–9]. SADI-S is currently considered a safe and effective treatment for severe obesity and related complications by the International Federation for the Surgery of Obesity and Metabolic Disorders (IFSO) with acceptable early complication rates (5.3%) and late complications mostly related to patients’ nutritional status (e.g. severe protein energy malnutrition, iron deficiency) [10].

Still, there is a wide range in postoperative outcomes following SADI-S, and potential factors contributing to this variation may include common channel (CC) length, primary versus secondary procedure, and indication for secondary SADI-S (e.g. planned second stage, recurrent weight gain, suboptimal initial response) [11]. Furthermore, only few studies consider the implications of SADI-S on patient-reported outcomes, such as health-related quality of life (HRQoL) and gastro-intestinal symptoms including gastroesophageal reflux disease (GERD) and constipation or diarrhea [12].

The primary aim of this study is to compare mid-term outcomes on weight loss, nutrient status, and patient-reported HRQoL and gastro-intestinal symptoms up to 5 years after primary versus secondary SADI-S. Secondary aims are to explore differences in weight loss outcomes between different indications for secondary SADI-S and between different CC lengths (≤ 250 cm or > 250 cm).

## Methods

### Study Design and Population

All adult patients who underwent primary or secondary SADI-S between June 2015 and February 2019 at one of four participating centers in the Netherlands (Rijnstate Hospital, Arnhem; Catharina Hospital, Eindhoven; St. Antonius Hospital, Nieuwegein; Dutch Obesity Clinic, The Hague) were included in this study. Exclusion criteria were known pregnancy during follow-up, malnutrition due to other causes (e.g. malignancies, alcoholism), previous metabolic bariatric surgery (other than laparoscopic adjustable banding or sleeve gastrectomy) and loss to follow-up directly after SADI-S.

**Fig. 1:**
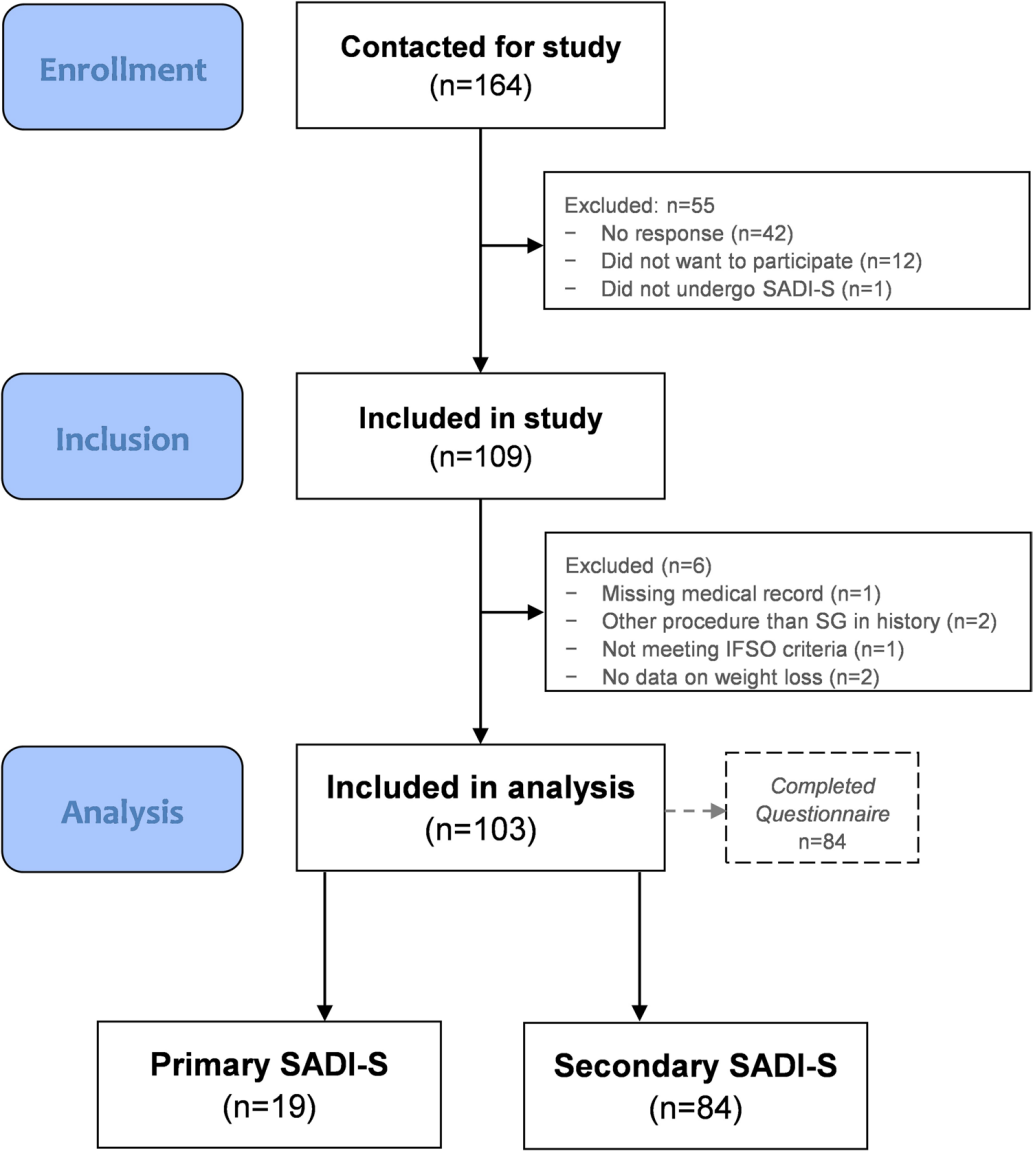
Flowchart patient selection and inclusion population

Patients were contacted by email and had the opportunity to digitally consent to either record-based research only (retrospective cohort) or to record-based research supplemented with additional questionnaires (cross-sectional). Of the 164 patients that were contacted for the study, 109 (66%) agreed to participate of whom six were excluded because of missing medical records (*n* = 1), history of other metabolic bariatric procedures (*n* = 2), not meeting IFSO criteria at the time of SADI-S (*n* = 1) or missing data on weight loss (*n* = 2). This resulted in a total study population of 103 participants (Fig. 1). Additional questionnaires on HRQoL and gastro-intestinal symptoms were completed by *n* = 84 at a median follow-up of 32 months after SADI-S.

The study protocol was reviewed and approved by the institutional review boards of the participating centers and all participants agreed to participate in this study.

All patients were divided into two groups: primary SADI-S or secondary SADI-S. Patients in the secondary SADI-S group were subsequently categorized into one of the following three indications for secondary SADI-S; 1: persistent ≥ class 2 obesity following SG (defined as patients that had a preoperative BMI ≥ 35 kg/m^2^ pre SADI-S and therefore still met IFSO-criteria for metabolic bariatric surgery (MBS) after SG), 2: suboptimal clinical response (defined as TWL < 20% at nadir after SG) or 3: recurrent weight gain (defined as a 30% increase of total weight lost in kilograms from nadir after SG). Additionally, the total cohort was divided into two groups based on their CC length after SADI-S: ≤ 250 cm or > 250 cm. In four cases, data on CC length was missing.

### SADI-S Procedure and Follow-up

First, a SG is performed, either during the same procedure (primary SADI-S) or at a previous occasion (secondary SADI-S). In both primary and secondary SADI-S, SG was performed in a similar fashion. When performing the SG, the abdomen was insufflated with CO2 using a Verress needle up to a maximum pressure of 19 mmHg. The first trocar was placed using a Blunttip-trocar under sight of the Endo-eye laparoscope after which the other trocars and the liver retractor were placed. Access to the omental bursa was obtained via the large curvature of the stomach and the stomach and the omentum were separated. The fundus and the angle of His were dissected and subsequently the stomach was exposed 2–4 cm proximal of the pylorus. Next, a 40-French gastric tube was advanced up to the pylorus. A linear stapled sleeve gastrectomy (Echelon, Ethicon, Johnson&Johnson) was performed parallel to the gastric tube, sparing 4 cm of gastric antrum proximal to the pylorus.

To complete the SADI-S configuration, the stomach was held upwards to identify the pylorus and dissect the duodenum 3 cm distal to the pylorus. The duodenum was then transected with a linear stapler. From the ileocecal junction, the surgeon measured the ileum with 5-cm intervals up to a length of 200–300 cm, after which the point for anastomosis was marked. The designated CC limb was pulled cranially to be anastomosed with the proximal duodenal stump using a stapler and/or V-loc sutures according to the surgeon’s preference.

After discharge, patients received follow-up at the outpatient clinic for 5 years. Standard laboratory blood tests were performed at least twice during the first year and yearly afterwards. Evaluated laboratory parameters included hemoglobin, ferritin, folic acid, vitamins A, B1, B6, B12 and D, calcium, parathyroid hormone (PTH) and albumin. Nutrient supplementation was advised to all patients, consisting of a (weight loss surgery) multivitamin supplement with additional calcium/vitamin D supplementation daily.

### Data Collection

Data on patient characteristics (gender, age, anthropometrics and presence of obesity-related complications), the SADI-S procedure (timing, duration, CC length, hospital stay, complications), weight loss and nutrient status were retrospectively extracted from medical records.

Complications were divided into short-term complications (≤ 30 days) and long-term complications (> 30 days), and were scored using the Clavien-Dindo classification [13]. Weight loss was defined as percentage TWL (weight loss at follow-up divided by preoperative weight). A nutrient deficiency was defined as a serum level below the local reference value at the time of blood collection.

HRQoL was measured using the BODY-Q questionnaire using the domains body image, physical function, psychological function, sexual well-being and social function. Scores in the BODY-Q questionnaires range from 0 to 100 with 0 being the worst score and 100 the best [14]. Gastro-intestinal symptoms that were evaluated included GERD, constipation and diarrhea. Complaints of GERD were assessed using the GERD-Health Related Quality of Life Questionnaire (GERD-HRQoL), which contains ten questions concerning reflux and dysphagia. A total score of 0 is equal to no complaints and a score of 50 to very severe complaints [15]. Information on defecation pattern was retrieved via the Fecal Score (FS). The FS is based on fecal frequency, fecal consistency and hinder in daily life. Fecal frequency and consistency were scored on a 5-point scale. Hinder in daily life was scored on a 6-point scale [16].

### Data Analysis

Data are reported as mean ± standard deviation (normal distribution) or as median [Q1, Q3] (non-normal distribution) for continuous variables, and as frequency (percentage) for categorical variables, unless stated otherwise.

Differences in patient characteristics between different indications for SADI-S and CC-length were compared using independent samples t-tests, Mann–Whitney U tests and Chi-square tests for normal continuous data, non-normal continuous data and count data, respectively.

Differences in weight loss outcomes between the groups were analyzed using linear mixed-effects models. The crude model consisted of fixed effects for group (SADI-S indication or CC length), follow-up time (baseline, 6 mo, 1y, 2y, 3y, 4, 5y) and their interaction term, plus a random effect for participants. Time entered the model as a repeated measure using an autoregressive covariance structure. Log-likelihood ratio tests were performed to explore potential confounders. Final models included gender and CC length (≤ 250 cm/> 250 cm) as confounder for weight loss per SADI-S indication and gender and SADI-S indication (primary/secondary) as confounder for weight loss per CC length. Results are presented as estimated marginal means and 95% confidence intervals (95% CI). Means and standard deviations of the original data at the different follow-up times can be found in Tables S1a and S1b.

The prevalence of nutrient deficiencies during follow-up was compared between the groups using Chi-Square tests or Fisher’s Exact test (if > 20% of expected counts were less than 5). Differences in HRQoL and patient-reported symptoms were analyzed using independent samples t-tests, and Chi-Square tests, respectively.

All statistical analyses were performed using IBM SPSS Statistics 29 for Windows (IBM Corp., Armonk USA). A two-sided *p*-value below 0.05 was considered statistically significant.

## Results

The majority of the total study population was female (81%) with a mean age of 43.3 ± 11.1 years (Table 1). A total of 19 patients (18%) underwent SADI-S as a primary procedure and 84 patients (82%) as a secondary procedure after SG: 49 (58%) because of persistent ≥ class 2 obesity following SG, 15 (18%) due to suboptimal clinical response and 20 (24%) because of recurrent weight gain.

**Table 1:** General patient characteristics for the total cohort, and primary and secondary SADI-S

	Total cohort (*n* = 103)	Primary SADI-S (*n* = 19)	Secondary SADI-S (*n* = 84)	*p* value
**Gender** (female)	83 (80.6)	14 (73.3)	69 (82.1)	0.52
**Age** (years)	43.3 ± 11.1	45.9 ± 12.7	42.7 ± 10.7	0.26
**Interval between SG and SADI-S** (months)	-	NA	34.5 [23.0, 58.5]	NA
**Duration of SADI-S procedure** (min)^a^	73.5 [60, 94]	104 [90, 114]	69 [59, 88]	** < 0.001**
**Length of common channel** (cm)^b^	250 [250, 300]	300 [250, 300]	250 [250, 250]	** < 0.001**
**Hospital stay** (days)	2.0 [1.0, 2.0]	2.0 [2.0, 2.0]	1.0 [1.0, 2.0]	**0.006**

Data represented as mean ± standard deviation, median [Q1, Q3] or frequency (%)

*SG* sleeve gastrectomy*, SADI-S* single-anastomosis duodeno-ilial bypass with sleeve gastrectomy

^a^ missing for *n* = 5

^b^ missing for *n* = 4

The median interval between SG and SADI-S for patients undergoing SADI-S as a second step procedure was 34.5 [23.0, 58.5] months. Median duration of the procedure, length of CC and hospital stay were shorter in the secondary SADI-S group compared to the primary SADI-S group (*p* < 0.05; Table 1).

Completed follow-up rates were 91% at 1 year, 73% at 2 years, 69% at 3 years, 62% at 4 years, and 53% at 5 years. All other patients were lost to follow-up.

### Complications

A total of six complications (5.8%) were registered in the first 30 days postoperatively (Table 2). Five patients had a Clavien-Dindo grade III complication: one due to jejnunal perforation, two due to anastomotic leakage, one due to anastomotic bleeding and one due to an intra-abdominal abscess. All < 30 day complications occurred in the secondary SADI-S group.

**Table 2:** Short- and long-term complications after SADI-S

Complications	*n* (%)
Short term (< 30 days)	6 (5.8)
Clavien-Dindo I	0 (0)
Clavien-Dindo II	1 (0.9)
Deep venous thrombosis	1 (0.9)
Clavien-Dindo III	5 (4.8)
Jejunal perforation	1 (0.9)
Anastomotic leakage	2 (1.9)
Anastomotic bleeding	1 (0.9)
Intra-abdominal abscess	1 (0.9)
Clavien-Dindo IV	0 (0)
Clavien-Dindo V	0 (0)
Long term (> 30 days)	7 (6.7)
Clavien-Dindo I	1 (0.9)
Surgical site infection	1 (0.9)
Clavien-Dindo II	0 (0)
Clavien-Dindo III	6 (5.8)
Revision cc length	4 (3.8)
Conversion to RYGB	1 (0.9)
Internal herniation	1 (0.9)
Clavien-Dindo IV	0 (0)
Clavien-Dindo V	0 (0)

Data are represented as valid frequency (%)

Seven long term complications (6.7%) were registered, six of these where Clavien-Dindo grade III complications. One patient underwent revisional surgery due to excessive weight loss, one due to invalidating diarrhea and two due to insufficient weight loss, one was converted to a RYGB because of a relative stenosis in the gastric pouch and one internal herniation was reported. One CD II complication occurred in the primary SADI-S group after > 30 days and all other complications occurred in the secondary SADI-S group. There were no complications with a Clavien-Dindo classification of IV or V.

### Weight Loss

For patients who underwent SADI-S as a secondary procedure, mean BMI decreased from 55.6 ± 8.1 kg/m^2^ before SG to 40.1 ± 6.4 kg/m^2^ at nadir (median 12 [12, 24] months post-SG). Maximum TWL at nadir was 27.2 ± 8.5% and 81% had reached a TWL of ≥ 20% after SG.

Before SADI-S, weight and BMI were comparable between the primary and secondary SADI-S group (Table 3). During follow-up after SADI-S, TWL was significantly higher in the primary SADI-S group compared to the secondary SADI-S group at all time points (*p* < 0.001 for all), resulting in a nadir TWL of 37.2 ± 5.8% after primary SADI-S (at median 24 [12, 36] months) and 21.9 ± 10.6% after secondary SADI-S (at median 12 [7.5, 36] months). At 5 years of follow-up, TWL was 34.8 (29.8–39.9)% for primary SADI-S vs 15.9 (13.0–18.9)% for secondary SADI-S (*p* < 0.001).

**Table 3:** Weight outcomes over time before and after primary and secondary SADI-S

		*n*	Total cohort	*n*	Primary SADI-S	*n*	Secondary SADI-S	*p* value^a^
**Weight** (kg)								** < 0.001**
	Before SADI-S	*99*	128.9 (123.3–134.6)	*19*	126.1 (116.7–135.5)	*80*	131.8 (126.0–137.6)	0.30
	6 months	*93*	102.7 (97.1–108.3)	*19*	93.5 (84.1–102.9)	*74*	111.8 (106.0–117.6)	**0.001**
	1 year	*90*	96.3 (90.6–101.9)	*17*	85.2 (75.7–94.6)	*73*	107.4 (101.5–113.2)	** < 0.001**
	2 years	*72*	95.0 (89.3–100.6)	*17*	82.6 (73.1–92.0)	*55*	107.4 (101.5–113.3)	** < 0.001**
	3 years	*68*	96.8 (91.1–102.5)	*15*	84.7 (75.1–94.2)	*53*	108.9 (102.9–114.8)	** < 0.001**
	4 years	*61*	98.2 (92.4–104.0)	*14*	87.0 (77.4–96.7)	*47*	109.3 (103.3–115.4)	** < 0.001**
	5 years	*53*	98.3 (92.3–104.4)	*10*	86.3 (76.1–96.4)	*43*	110.4 (104.2–116.6)	** < 0.001**
**BMI** (kg/m^2^)								** < 0.001**
	Before SADI-S	*99*	42.3 (40.4–44.1)	*19*	41.0 (37.8–44.1)	*80*	43.6 (41.6–45.5)	0.16
	6 months	*93*	33.2 (31.3–35.1)	*19*	29.7 (26.6–32.9)	*74*	36.7 (34.7–38.6)	** < 0.001**
	1 year	*90*	31.0 (29.2–32.9)	*17*	26.9 (23.7–30.0)	*73*	35.2 (33.2–37.1)	** < 0.001**
	2 years	*72*	30.6 (28.7–32.4)	*17*	26.0 (22.8–29.1)	*55*	35.1 (33.2–37.1)	** < 0.001**
	3 years	*68*	31.2 (29.3–33.1)	*15*	26.7 (23.5–29.9)	*53*	35.7 (33.7–37.6)	** < 0.001**
	4 years	*61*	31.7 (29.7–33.6)	*14*	27.6 (24.4–30.8)	*47*	35.7 (33.7–37.8)	** < 0.001**
	5 years	*53*	31.7 (29.7–33.7)	*10*	27.4 (24.0–30.7)	*43*	36.1 (34.0–38.1)	** < 0.001**
**TWL** (%)								** < 0.001**
	6 months	*93*	21.9 (19.3–24.5)	*19*	29.0 (24.7–33.4)	*74*	14.8 (12.2–17.3)	** < 0.001**
	1 year	*90*	26.9 (24.3–29.4)	*17*	35.6 (31.3–40.0)	*73*	18.1 (15.5–20.7)	** < 0.001**
	2 years	*72*	28.0 (25.4–30.6)	*17*	37.7 (33.4–42.1)	*55*	18.3 (15.6–21.0)	** < 0.001**
	3 years	*68*	26.6 (23.9–29.2)	*15*	36.0 (31.5–40.4)	*53*	17.2 (14.4–19.9)	** < 0.001**
	4 years	*61*	25.4 (22.7–28.2)	*14*	34.2 (29.7–38.8)	*47*	16.6 (13.8–19.5)	** < 0.001**
	5 years	*53*	25.4 (22.4–28.3)	*10*	34.8 (29.8–39.9)	*43*	15.9 (13.0–18.9)	** < 0.001**

Data represented as estimated marginal mean (95% CI)

*SADI-*S single-anastomosis duodeno-ilial bypass with sleeve gastrectomy*, BMI* body mass index, *TWL* total weight loss

^a^Model adjusted for gender (male/female) and CC length (≤ 250 cm/> 250 cm)

**Fig. 2:**
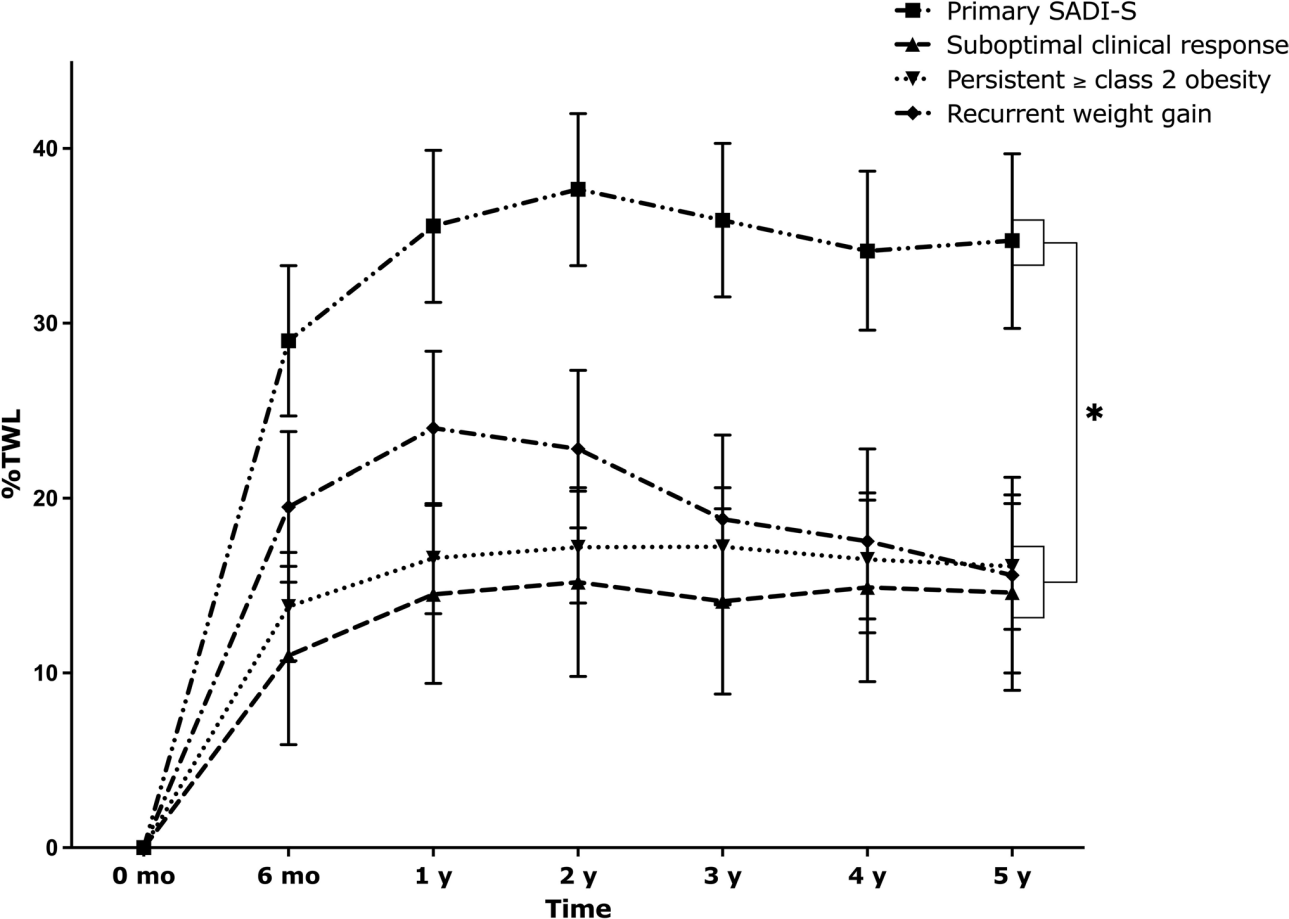
TWL (%) after SADI-S for the total study population and per SADI-S indication. Data are presented as estimated marginal means (95% CI)

When subdividing the secondary SADI-S group into the three different indications, TWL in the recurrent weight gain group was significantly higher compared to the suboptimal clinical response group at 6 months (19.5 (15.2–23.8)% vs 11.0 (5.9–16.1)%, *p* = 0.049) and 1 year (24.0 (19.6–28.4)% vs 14.5 (9.4–19.6)%, *p* = 0.02) after SADI-S (Fig. 2). Additionally, TWL in this group was higher than in the persistent ≥ class 2 obesity group at 1 year (16.6 (13.4–20.0)%, *p* = 0.02). Thereafter, weight loss was similar between the three groups.

**Fig. 3:**
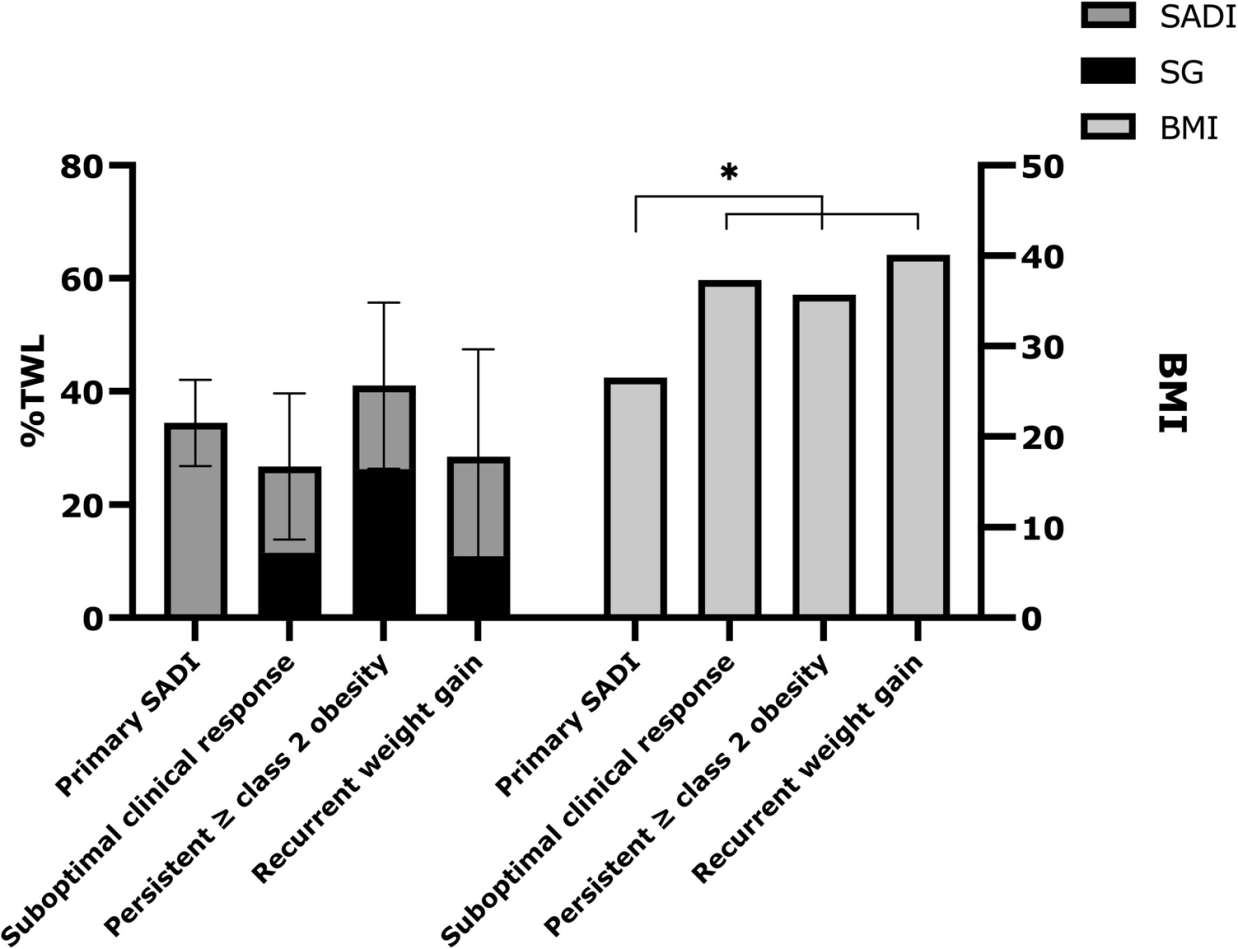
TWL (%) and BMI per SADI-S indication at 5 years postoperatively, including TWL after SG before the secondary SADI-S procedure

When weight loss of the SG was taken into account in the secondary SADI group, total TWL at 5 years in this group (32.0 ± 14.9%) was comparable to the primary SADI-S group (34.4 ± 7.6%), although BMI at 5 years was still lowest in the primary SADI-S group (Fig. 3).

### Nutrient Status

The prevalence of nutrient deficiencies for ferritin (12–20%), folic acid (19–27%), vitamin D (24–31%) and zinc (57–76%) as well as anemia (19–26%) and elevated PTH levels (61–77%) increased after SADI-S (Table 4). However, vitamin B12 deficiency decreased from 47% before SADI-S to 3–13% during follow-up. Deficiencies for vitamins A, B1, B6 and calcium, and low albumin levels were not common before and after SADI-S (Table 4).

**Table 4:** Nutrient deficiencies over time before and after SADI-S

	Critical range	Before SADI-S^a^	After SADI-S		
			*1 year*	*3 years*	*5 years*
**Anemia**	F: < 7.5 mmol/L M: < 8.5 mmol/L	6/93 (6.5)	22/85 (25.9)	14/55 (25.5)	7/37 (18.9)
**Ferritin**	< 20 µg/L	8/76 (10.5)	9/78 (11.5)	10/49 (20.4)	6/36 (16.7)
**Iron**	< 10 µmol/L	6/36 (16.7)	4/35 (11.4)	7/23 (30.4)	1/15 (6.7)
**Folic acid**	< 10 nmol/L	12/76 (15.8)	21/79 (26.6)	10/48 (20.8)	7/36 (19.4)
**Vitamin A**	< 0.7 µmol/L	0/3 (0.0)	0/36 (0.0)	0/21 (0.0)	0/8 (0.0)
**Vitamin B**_**1**_	< 70 nmol/L	0/36 (0.0)	1/56 (1.8)	1/31 (3.2)	0/22 (0.0)
**Vitamin B**_**6**_	< 35 nmol/L	0/36 (0.0)	0/58 (0.0)	0/31 (0.0)	0/23 (0.0)
**Vitamin B**_**12**_	< 295 pmol/L	36/76 (47.4)	9/82 (11.0)	6/47 (12.8)	1/34 (2.9)
**Vitamin D**	< 50 mmol/L	16/77 (20.8)	26/84 (31.0)	15/53 (28.3)	9/37 (24.3)
**Calcium**	< 2.15 mmol/L	0/59 (0.0)	7/85 (8.2)	7/53 (13.2)	1/37 (2.7)
**PTH**^**b**^	> 7 pmol/L	16/76 (21.1)	49/81 (60.5)	34/49 (69.4)	27/35 (77.1)
**Albumin**	< 35 g/L	0/49 (0.0)	6/73 (8.2)	3/52 (5.8)	1/34 (2.9)
**Zinc**	< 10 µmol/L	1/4 (25.0)	25/33 (75.8)	15/22 (68.2)	4/7 (57.1)

Data are represented as valid frequency (%)

^*F*^ female, ^*M*^ male, *PTH* parathyroid hormone

^a^ Only Hb was assessed before primary SADI-S

^b^ elevated PTH levels

There was no significant difference in nutrient status between primary and secondary SADI-S (data not shown).

### Health-Related Quality of Life and Gastro-Intestinal Symptoms

On a scale of 0–100, highest scores regarding HRQoL were found for the subscales ‘physical function’ (78.0 ± 20.4), ‘social function ‘ (68.9 ± 22.4) and ‘psychological function’ (62.9 ± 22.9) (Table 5).

**Table 5:** Health-related quality of life (BODY-Q) scores after SADI-S

	Total cohort (*n* = 84)	Primary SADI-S (*n* = 17)	Secondary SADI-S (*n* = 67)	*p* value	*Persistent ≥ class 2 obesity* *(n* = *40)*	*Suboptimal clinical response* *(n* = *13)*	*Recurrent weight gain* *(n* = *14)*
Body image	44.1 ± 24.2	62.9 ± 24.6	39.3 ± 21.7	** < 0.001**	40.6 ± 22.3	37.9 ± 20.7	36.9 ± 22.3
Physical function	78.0 ± 20.4	90.2 ± 14.3	75.0 ± 20.7	**0.001**	77.2 ± 20.0	70.9 ± 21.4	72.3 ± 22.6
Psychological function	62.9 ± 22.9	74.2 ± 21.8	60.0 ± 22.5	**0.02**	61.6 ± 23.4	57.4 ± 25.2	58.0 ± 17.9
Sexual well-being	48.9 ± 24.4	57.9 ± 20.8	46.6 ± 24.9	0.09	51.9 ± 23.0	38.4 ± 23.8	39.1 ± 28.7
Social function	68.9 ± 22.4	80.3 ± 21.8	66.0 ± 21.8	**0.02**	69.8 ± 22.7	63.5 ± 17.3	57.9 ± 21.6

Data represented as mean ± standard deviation

BODY-Q scores were analyzed at a median of 32 months post SADI-S

When comparing HRQoL, patients who underwent primary SADI-S had significant higher scores for ‘body image’, ‘physical function’, ‘psychological function’ and ‘social function’ compared to patients who underwent a secondary SADI-S (*p* < 0.05 for all; Table 5). For the sub-indications of secondary SADI- S, HRQoL was comparable between the groups for all domains of the BODY-Q questionnaires (*p* > 0.05 for all).

The severity of GERD symptoms after SADI-S was low with a median score of 2 [0, 10], and a maximum reported score of 34. Still, 43 patients (51%) reported using a proton pump inhibitor (PPI) at a median of 32 months postoperatively.

Most patients reported a defecation frequency of more than two times daily (48%) with a median frequency of 4 [3, 5] times daily after SADI-S. Consistency was mostly pulpy and soft (71%), and 26% of patients reported never experiencing hinder in daily life due to their defecation frequency or consistency, whereas 19% experienced this on a daily basis. There were no differences in fecal score between primary and secondary SADI-S (data not shown).

### Common Channel Length

Total range for CC length was 120–300 cm, with CC length ≤ 250 cm in 66 patients (67%) and > 250 cm in 33 patients (33%). Both groups significantly differed with respect to age, preoperative weight and BMI and length of hospital stay (Table S2).

**Fig. 4:**
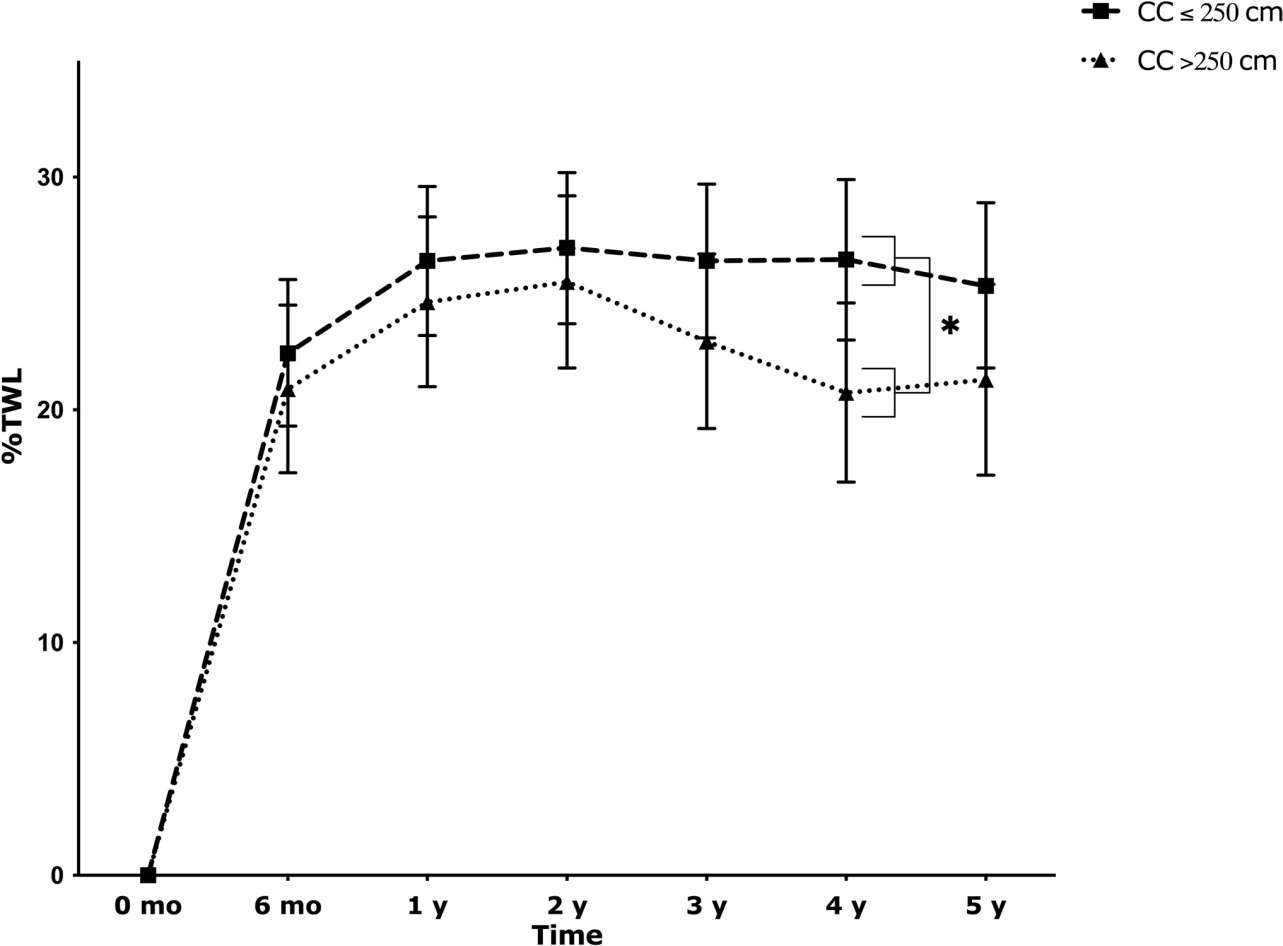
TWL (%) after SADI-S per CC length. Data are presented as estimated marginal means (95% CI)

During follow-up after SADI-S, TWL tended to be higher in the CC ≤ 250 cm group compared to the > 250 cm group, resulting in 25.3 (21.8–28.9)% vs 21.3 (17.2–25.4)% TWL at 5 years of follow-up (*p* = 0.12; Table 6, Fig. 4).

**Table 6:** Weight outcomes over time between SADI with CC ≤ 250 cm and > 250 cm

		*n*	Total cohort	*n*	CC ≤ 250 cm	*n*	CC > 250 cm	*p* value^a^
**Weight** (kg)								0.70
	Before SADI-S	*99*	125.6 (120.0–131.1)	*66*	131.8 (124.9–138.7)	*33*	119.4 (111.9–126.9)	**0.01**
	6 months	*93*	103.2 (97.6–108.7)	*62*	109.3 (102.4–116.2)	*31*	97.0 (89.5–104.5)	**0.01**
	1 year	*90*	98.1 (92.6–103.7)	*61*	103.8 (96.9–110.7)	*29*	92.5 (84.9–100.0)	**0.02**
	2 years	*72*	97.5 (91.8–103.1)	*45*	103.5 (96.5–110.5)	*27*	91.4 (83.8–99.0)	**0.01**
	3 years	*68*	99.4 (93.8–105.1)	*43*	104.3 (97.2–111.3)	*25*	94.6 (86.9–102.3)	**0.05**
	4 years	*61*	100.6 (94.9–106.4)	*39*	104.2 (97.0–111.4)	*22*	97.1 (89.2–104.9)	0.15
	5 years	*53*	101.2 (95.3–107.1)	*34*	105.7 (98.4–113.1)	*19*	96.6 (88.5–104.7)	0.07
**BMI** (kg/m^2^)								0.64
	Before SADI-S	*99*	41.1 (39.3–43.0)	*66*	42.6 (40.3–44.9)	*33*	39.7 (37.2–42.2)	0.07
	6 months	*93*	33.4 (31.5–35.2)	*62*	34.9 (32.6–37.2)	*31*	31.9 (29.3–34.4)	0.05
	1 year	*90*	31.7 (29.8–33.5)	*61*	33.1 (30.8–35.4)	*29*	30.3 (27.8–32.8)	0.08
	2 years	*72*	31.4 (29.5–33.3)	*45*	32.9 (30.6–35.2)	*27*	29.9 (27.4–32.4)	0.06
	3 years	*68*	32.1 (30.2–34.0)	*43*	33.2 (30.8–35.5)	*25*	31.0 (28.4–33.6)	0.18
	4 years	*61*	32.5 (30.6–34.4)	*39*	33.1 (30.7–35.5)	*22*	31.9 (29.3–34.5)	0.47
	5 years	*53*	32.6 (30.6–34.6)	*34*	33.6 (31.1–36.0)	*19*	31.7 (29.0–34.4)	0.27
**TWL** (%)								0.49
	6 months	*93*	21.7 (19.1–24.2)	*62*	22.4 (19.3–25.6)	*31*	20.9 (17.3–24.5)	0.49
	1 year	*90*	25.5 (22.9–28.1)	*61*	26.4 (23.2–29.6)	*29*	24.6 (21.0–28.3)	0.44
	2 years	*72*	26.2 (23.6–28.9)	*45*	27.0 (23.7–30.2)	*27*	25.5 (21.8–29.2)	0.52
	3 years	*68*	24.7 (22.0–27.3)	*43*	26.4 (23.1–29.7)	*25*	22.9 (19.2–26.7)	0.14
	4 years	*61*	23.6 (20.8–26.4)	*39*	26.5 (23.0–29.9)	*22*	20.7 (16.9–24.6)	**0.02**
	5 years	*53*	23.3 (20.4–26.2)	*34*	25.3 (21.8–28.9)	*19*	21.3 (17.2–25.4)	0.12

Data represented as estimated marginal mean (95% CI)

*SADI-*S single-anastomosis duodeno-ilial bypass with sleeve gastrectomy*, BMI* body mass index, *TWL* total weight loss

^a^Model adjusted for gender and SADI indication (primary/secondary)

There was no significant difference in the prevalence of nutrient deficiencies between the CC groups after SADI-S (Table S3). However, anemia and folic acid deficiencies at 1 year post-SADI tended to be more prevalent in the CC ≤ 250 cm group compared to the CC > 250 cm group (32% vs 15%, *p* = 0.11; 33% vs 17%, *p* = 0.17, respectively). Furthermore, deficiencies for iron (15–22% vs 0–8%, *p* > 0.05) and vitamin D (29–32% vs 13–17%, *p* > 0.05) tended to be more common in the CC ≤ 250 cm group at 3 and 5 years of follow-up. Severe protein energy malnutrition, presented as low albumin levels, were also only observed in patients with a CC ≤ 250 cm (4–12%).

**Fig. 5:**
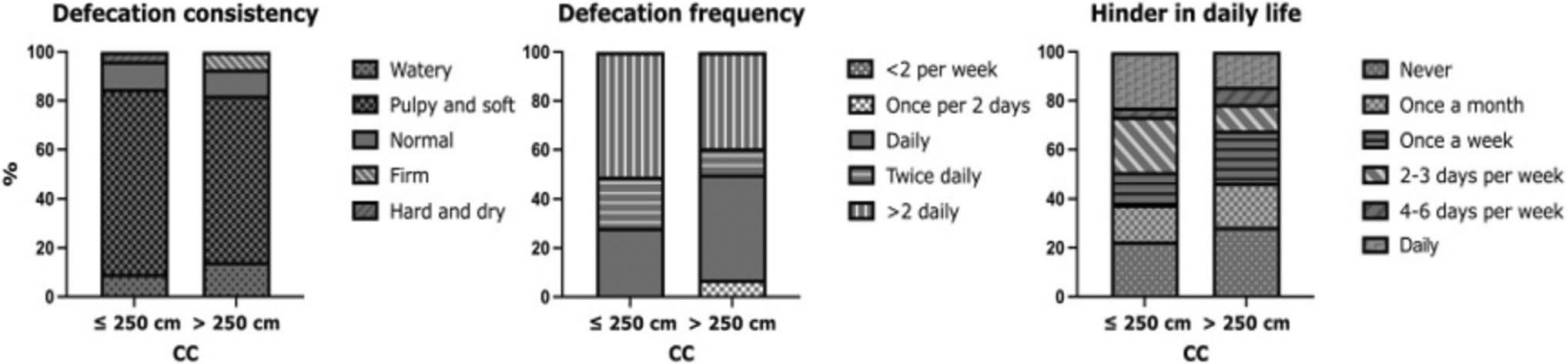
Fecal score after SADI-S according to CC length

Patients with a CC length of ≤ 250 cm tended to defecate more often with 50% of patients reporting more than two times per day (median 4 times) compared to 39% in the CC > 250 cm group (median 3 times, *p* = 0.09) (Fig. 5). Fecal consistency and hinder in daily life were similar, and there were no differences in HRQoL between the CC groups (data not shown).

## Discussion

The aim of this study was to describe mid-term outcomes on weight loss, nutrient status and patient-reported HRQoL and gastro-intestinal symptoms up to 5 years after primary versus secondary SADI-S, and to explore differences in these outcomes between different indications for secondary SADI-S (persistent ≥ class 2 obesity, suboptimal clinical response, recurrent weight gain) and between different CC lengths (≤ 250 cm or > 250 cm).

A significantly higher mean TWL of 34.8 (29.8–39.9)% versus 15.9 (13.0–18.9)% at five years was found for primary SADI-S compared to secondary SADI-S. In a recent study by Osorio et al. [17], TWL was 27.3% for primary SADI-S and 24.4% for secondary SADI-S at 5 years postoperatively. The discrepancy between these findings could be attributed to the fact that %TWL in the study of Osorio et al. was measured 5 years from the primary procedure whilst %TWL in our cohort is reported at 5 years post-secondary SADI-S. Furthermore, our findings are also not in line with the systematic review by Esparham et al. [18], who reported a %TWL for primary SADI-S of 38.8% and 37.0% for secondary SADI-S at 5 years postoperatively. We do not have a valid explanation for the discrepancies between our results and the study by Esparham et al. Similar studies comparing primary versus secondary MBS procedures were performed for OAGB and RYGB [19, 20]. Both studies found a higher %TWL in primary procedures compared to secondary procedures, which is in line with our findings in SADI-S.

When the initial weight loss of the SG procedure is taken into account for patients who underwent SADI-S as a second step procedure, weight loss outcomes at 5 years were similar for primary and secondary SADI-S (34.4% vs 32.0% TWL). This indicates that both procedures are equally effective in terms of weight loss. Yet, BMI at 5 years was still lowest in the primary SADI-S group. This can be attributed to several factors, for example: a lower initial BMI, a relatively long median interval to the secondary procedure (34 months) leading to some amount of recurrent weight gain and thus a suboptimal result of the combined procedures. Also, part of the secondary procedures were performed due to a suboptimal clinical response following SG, which might indicate a lower response to metabolic surgery overall.

Interestingly, the recurrent weight loss group showed higher %TWL until 1 year postoperatively, compared to the suboptimal clinical response and persistent ≥ class 2 obesity group, after which it decreased to similar weight loss for all groups. These variations in weight loss patterns might be explained by individual differences in demographic, biological, psychological and behavioral determinants of weight loss [21].

Although not statistically different for all time points, we also found a trend towards higher weight loss for CC length ≤ 250 cm compared to > 250 cm. An explanation for this difference is that CC length ≤ 250 cm per definition result in more malabsorption. To our knowledge, there are no previous studies that compare differences in TWL following SADI-S according to CC lengths.

When assessing HRQoL, patients who underwent secondary SADI-S had significant lower scores for ‘body image’, ‘physical function’, ‘psychological function’ and ‘social function’ compared to patients who underwent a primary SADI-S, although overall HRQoL was acceptable. These BODY-Q scores are comparable to the scores that Makarawung et al. found in their multicenter, cross-sectional studies in patients that underwent RYGB or SG more than 3 years postoperatively [22]. Dalaei et al. investigated the normative scores in patients that did not undergo any type of MBS [23]. On the domains of psychological and physical function, scores found in our study were comparable to their findings: 62.9 vs 61.5 for psychological function and 78.0 vs 81.2 for physical function, respectively. Patients that underwent MBS did score differently on the domains of social wellbeing, sexual wellbeing and body image compared to the general population: 68.9 vs 59.5 for social wellbeing, 48.9 vs 67.0 for sexual wellbeing and 44.1 vs 53.5 for body image. Surprisingly, patients in our cohort that underwent SADI-S as a secondary procedure scored significantly lower on all BODY-Q domains, except for sexual wellbeing. We hypothesize that this difference can be attributed to the fact that patients who underwent SADI-S as a secondary procedure had their primary procedure (SG) earlier on in time. As shown by the study of Makarawung et al. [22], BODY-Q scores tend to decline over time in patients that underwent MBS. Admella et al.[12] analyzed patient-reported outcomes and quality of life after SADI-S using the SF- 36 physical and mental components. Their study showed improvement on both the physical and mental components at 3 years postoperatively compared to preoperatively. In our study, the BODY-Q scores were only measured at a median of 32 months post-SADI-S and therefore a comparison with preoperative data was not possible.

Our study showed a low prevalence of GERD and low scores in the GERD-HRQoL questionnaire which is comparable to the currently available literature [12]. However, almost half of the patients used PPIs at a median of 32 months postoperatively. It is unknown why PPI usage is this high and if this influenced the results in our study on the reported prevalence of GERD. However, GERD symptoms are known to increase after SG in roughly 20% of patients [24] and a study by Salminen et al. reported PPI use in 64% of patients at ten years follow up post- SG [25]. It can therefore be hypothesized that (chronic) PPI use is already high pre SADI-S and that patients have relatively low complaints of GERD due to using PPIs.

Most patients reported a defecation frequency of more than two times daily (48%), and consistency was mostly pulpy and soft (71%). Admella et al. [12] used the Bristol stool chart to evaluate the consistency of stools post-SADI-S. Although the fecal score and Bristol stool chart are not directly comparable, a Bristol score ≥ 5 could be compared to a defecation consistency that is described as watery in the fecal score questionnaire. Admella et al. found a higher percentage of patients having diarrhea at 2–3 years postoperatively compared to our results (25.4% vs 11.0%).

Patients with a CC length of ≤ 250 cm did not report different defecation patterns compared to patients with a CC > 250 cm, which may be attributed to the limited range of CC lengths in our study. However, they did tend to defecate more often with 50% of patients reporting more than two times per day (median 4 times) compared to 39% in the CC > 250 group (median 3 times).

Furthermore, anemia, iron deficiency and severe protein energy malnutrition tended to be more present in the CC ≤ 250 cm group. These findings also correspond to what is described in existing literature [26, 27]. Therefore, it is advised to use a CC length of at least 250 cm.

Complication rates in our study on both short- (≤ 30 days; 5.8%) and long-term (> 30 days; 6.7%) were acceptable and comparable to previously reported complication rates [28]. Interestingly, all < 30 day complications and six out of seven of the > 30 day complications occurred in the secondary SADI-S group. We hypothesize that this difference is due to the fact that revisional procedures are more difficult to perform. Franken et al. [29] also found a similar major complication rate of 6% (CD ≥ III), but a much higher minor complication rate of 36% (CD I-II) post SADI-S. This can be explained by the fact that nutrient deficiencies were scored as minor complications whereas we did not score deficiencies as minor complications.

## Strengths and Limitations

This study has several strengths. First, to our knowledge this is the first study that describes weight loss patterns in SADI-S for different indications and CC lengths. Second, the extensive description of health-related quality of life using the BODY-Q, fecal score, and GERD-HRQoL questionnaires gives important insights of patient reported outcomes following SADI-S.

This study also has some limitations. First, as many retrospective studies on MBS procedures, we also experienced a relatively high loss to follow-up rate. Despite all effort, we were only able to achieve a complete follow-up rate of 53% after 5 years resulting in a relatively small, heterogeneous study population. Outside of research om MBS procedures, loss to follow-up unfortunately also is a widespread challenging problem in daily clinical practice. Second, the total cohort consisted of 103 patients with only 19 patients undergoing SADI-S as a primary procedure, all coming from the same center. This may hamper a solid comparison of primary versus conversion SADI-S. Third, due to the retrospective design, general characteristics were not equally distributed between the different indications for SADI-S. Fourth, in case of secondary SADI-S after SG, information about whether or not a re-sleeve was performed during the SADI-S procedure was not reported. Fifth, potential influences of multivitamin supplementation use on nutrient status was not taken into account. Therefore, our outcomes on nutrient status and subsequent deficiencies following SADI-S should be interpreted with some caution. Finally, the cut-off for CC length at 250 cm is artificial and results may have been influenced due to two patients in the ≤ 250 cm group with a very short CC (respectively, 120 and 200 cm).

## Conclusion

Both primary and secondary SADI-S are safe procedures which result in good and durable weight loss outcomes up to 5 years postoperatively. It is imperative that common channel length should be at least 250 cm to prevent malnutrition and gastro-intestinal complaints. Furthermore, the implications of SADI-S procedures on health-related quality of life should not be overlooked, especially after secondary SADI-S. In future research, it is essential to broaden the focus beyond weight-related outcomes and to include patient-reported outcomes, allowing for a more comprehensive understanding of the impact on patients'daily lives.

## Supplementary Information

Below is the link to the electronic supplementary material.

Supplementary file1 (DOCX 346 KB)

Supplementary file2 (JPG 1.68 MB)

## Data Availability

The data that support the findings of this study are available from the corresponding author upon reasonable request.
