# Supplementary material for: Five year outcomes of primary and secondary Single-Anastomosis Duodeno-Ileal bypass with Sleeve gastrectomy (SADI-S)
Source: Obes Surg. 2025 May 9;35(6):2160–73. doi: 10.1007/s11695-025-07888-4 (PMC12130166; doi:10.1007/s11695-025-07888-4)
Supplement: Supplementary file 1 — Supplementary file1 (DOCX 346 KB) [file 11695_2025_7888_MOESM1_ESM.docx]

**Figure S1.** Flowchart patient selection and inclusion population per centre.


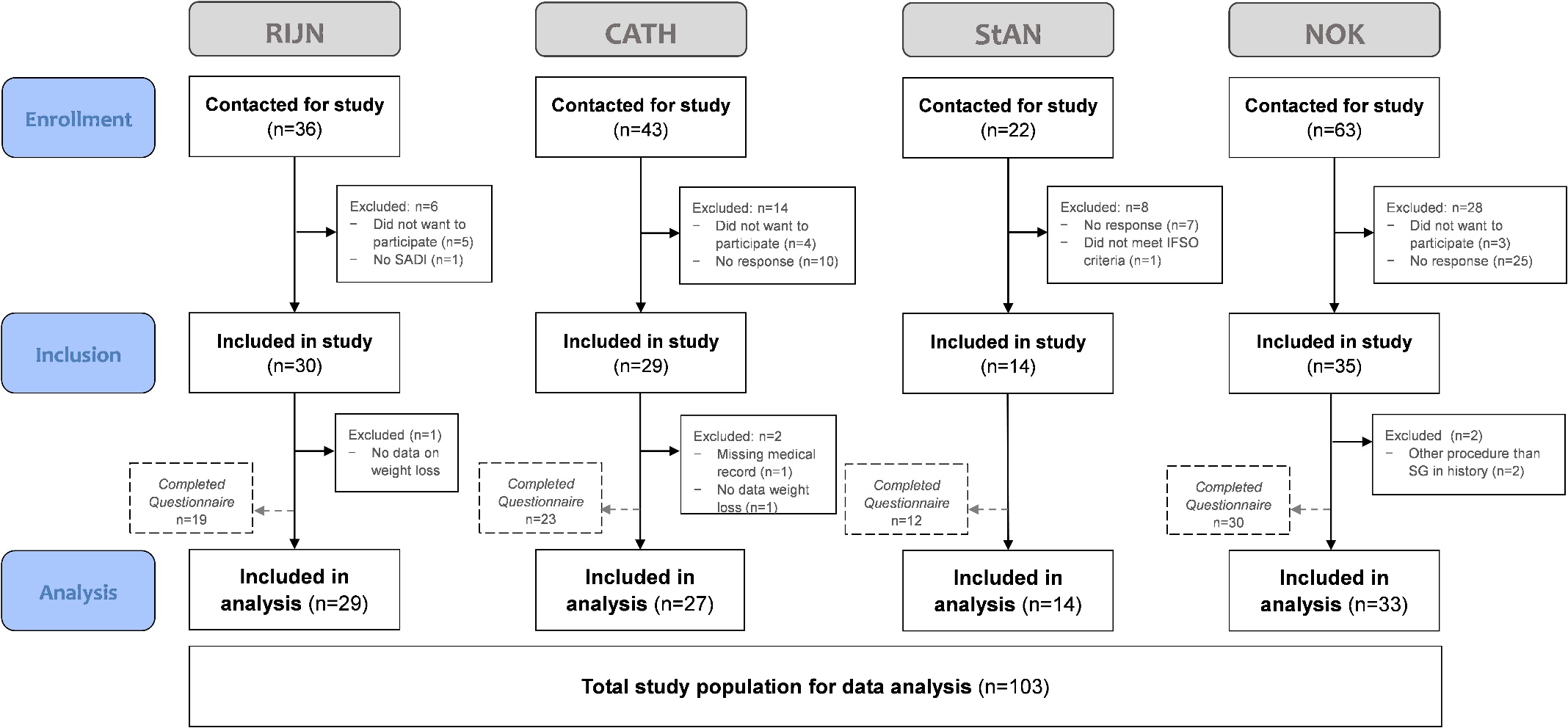


RIJN, Rijnstate Hospital, Arnhem; CATH ,Catharina Hospital Eindhoven; StAN, St. Antonius Hospital, Nieuwegein; NOK, Dutch Obesity Clinic, The Hague

**Table S1a.** Weight outcomes, split between indication for SADI-S

|  |  | ***n*** | **Total cohort** | ***n*** | **Primary SADI-S** | ***n*** | **Secondary SADI-S** | ***n*** | ***Persistent ≥ class 2 obesity*** | ***n*** | ***Suboptimal clinical response*** | ***n*** | ***Recurrent weight gain*** |
| --- | --- | --- | --- | --- | --- | --- | --- | --- | --- | --- | --- | --- | --- |
| **Weight** (kg) | Before SADI-S | *103* | 125.1 ± 23.9 | *19* | 118.1 ± 20.0 | *84* | 126.7 ± 24.5 | *49* | 122.3 ± 21.6 | *15* | 122.6 ± 16.9 | *20* | 140.5 ± 31.2 |
|  | 6 months | *97* | 102.9 ± 20.3 | *19* | 85.5 ± 17.3 | *78* | 107.2 ± 18.7 | *45* | 106.3 ± 18.8 | *14* | 107.0 ± 14.4 | *19* | 109.3 ± 21.7 |
|  | 1 year | *94* | 98.4 ± 20.7 | *17* | 79.0 ± 13.1 | *77* | 102.7 ± 19.7 | *45* | 100.6 ± 19.5 | *14* | 104.7 ± 13.9 | *18* | 105.6 ± 23.7 |
|  | 2 years | *75* | 97.5 ± 24.2 | *17* | 74.6 ± 12.9 | *58* | 104.2 ± 22.6 | *33* | 102.1 ± 22.3 | *9* | 103.4 ± 17.7 | *16* | 109.2 ± 26.0 |
|  | 3 years | *71* | 98.7 ± 22.2 | *15* | 77.8 ± 13.5 | *56* | 104.2 ± 20.7 | *31* | 99.5 ± 22.8 | *12* | 109.6 ± 12.6 | *13* | 110.5 ± 19.9 |
|  | 4 years | *63* | 98.2 ± 20.0 | *14* | 78.9 ± 14.0 | *49* | 103.7 ± 18.0 | *30* | 100.0 ± 18.8 | *11* | 110.8 ± 12.8 | *8* | 107.5 ± 15.3 |
|  | 5 years | *55* | 101.8 ± 24.9 | *10* | 77.1 ± 10 | *45* | 107.2 ± 24.0 | *26* | 103.3 ± 19.7 | *10* | 106.8 ± 20.8 | *9* | 119.1 ± 35.4 |
| **BMI** (kg/m^2^) | Before SADI-S | *103* | 43.4 ± 7.0 | *19* | 40.4 ± 6.0 | *84* | 44.1 ± 7.1 | *49* | 42.8 ± 6.7 | *15* | 43.4 ± 3.8 | *20* | 48.0 ± 8.7 |
|  | 6 months | *97* | 35.7 ± 6.7 | *19* | 29.1 ± 4.8 | *78* | 37.3 ± 6.1 | *45* | 36.7 ± 6.2 | *14* | 38.0 ± 3.6 | *19* | 38.1 ± 7.2 |
|  | 1 year | *94* | 34.1 ± 6.8 | *17* | 26.8 ± 3.4 | *77* | 35.8 ± 6.2 | *45* | 35.1 ± 6.3 | *14* | 37.0 ± 4.3 | *18* | 36.6 ± 7.3 |
|  | 2 years | *75* | 34.0 ± 8.0 | *17* | 25.5 ± 2.9 | *58* | 36.5 ± 7.2 | *33* | 36.0 ± 7.8 | *9* | 37.2 ± 5.5 | *16* | 37.1 ± 7.3 |
|  | 3 years | *71* | 34.3 ± 7.2 | *15* | 26.5 ± 3.2 | *56* | 36.4 ± 6.5 | *31* | 34.4 ± 6.3 | *12* | 38.4 ± 5.2 | *13* | 39.1 ± 7.0 |
|  | 4 years | *63* | 34.4 ± 6.8 | *14* | 27.1 ± 4.2 | *49* | 36.5 ± 5.8 | *30* | 35.0 ± 5.5 | *11* | 39.0 ± 5.2 | *8* | 38.7 ± 7.2 |
|  | 5 years | *55* | 35.0 ± 7.5 | *10* | 26.5 ± 3.1 | *45* | 36.9 ± 6.8 | *26* | 35.7 ± 5.9 | *10* | 37.3 ± 6.2 | *9* | 40.2 ± 9.2 |
| **TWL** (%) | Before SADI-S | *103* | - | *19* | - | *84* | - | *49* | - | *15* | - | *20* | - |
|  | 6 months | *97* | 17.6 ± 8.4 | *19* | 27.9 ± 5.0 | *78* | 15.1 ± 7.0 | *45* | 14.3 ± 5.9 | *14* | 11.6 ± 5.0 | *19* | 19.7 ± 8.6 |
|  | 1 year | *94* | 21.5 ± 11.1 | *17* | 34.3 ± 6.3 | *77* | 18.7 ± 9.9 | *45* | 17.2 ± 9.1 | *14* | 15.5 ± 9.0 | *18* | 25.0 ± 10.3 |
|  | 2 years | *75* | 22.7 ± 13.5 | *17* | 36.7 ± 6.3 | *58* | 18.6 ± 12.3 | *33* | 16.7 ± 12.2 | *9* | 15.0 ± 11.8 | *16* | 24.5 ± 11.5 |
|  | 3 years | *71* | 21.7 ± 13.6 | *15* | 35.2 ± 6.5 | *56* | 18.1 ± 12.7 | *31* | 19.3 ± 12.3 | *12* | 13.4 ± 11.6 | *13* | 19.3 ± 14.5 |
|  | 4 years | *63* | 20.0 ± 14.4 | *14* | 34.4 ± 5.8 | *49* | 15.4 ± 13.4 | *30* | 17.0 ± 13.2 | *11* | 11.8 ± 8.4 | *8* | 17.1 ± 19.6 |
|  | 5 years | *55* | 18.9 ± 15.7 | *10* | 34.4 ± 7.6 | *45* | 15.4 ± 14.9 | *26* | 14.8 ± 14.7 | *10* | 15.2 ± 12.9 | *9* | 17.5 ± 19.0 |

Data represented as mean ± standard deviation.

*SADI-S* single-anastomosis duodeno-ilial bypass with sleeve gastrectomy*, BMI* body mass index*, TWL* total weight loss

**Table S1b**. Weight outcomes, split between common channel lengths

|  |  | ***n*** | **Total cohort** | ***n*** | **CC ≤250 cm** | ***n*** | **CC >250cm** |
| --- | --- | --- | --- | --- | --- | --- | --- |
| **Weight** (kg) | Before SADI-S | *99* | 125.4 ± 24.1 | *66* | 131.2 ± 25.0 | *33* | 113.3 ± 16.9 |
|  | 6 months | *93* | 103.0 ± 20.6 | *62* | 108.8 ± 20.4 | *31* | 91.5 ± 15.7 |
|  | 1 year | *90* | 98.5 ± 21.1 | *61* | 103.4 ± 22.1 | *29* | 88.1 ± 14.7 |
|  | 2 years | *72* | 97.4 ± 24.6 | *45* | 104.9 ± 25.6 | *27* | 85.0 ± 17.0 |
|  | 3 years | *68* | 98.7 ± 22.6 | *43* | 103.9 ± 23.0 | *25* | 89.8 ± 19.0 |
|  | 4 years | *61* | 98.3 ± 20.2 | *39* | 100.8 ± 19.9 | *22* | 93.8 ± 20.4 |
|  | 5 years | *53* | 101.7 ± 25.2 | *34* | 105.4 ± 26.6 | *19* | 95.0 ± 21.7 |
| **BMI** (kg/m^2^) | Before SADI-S | *99* | 43.5 ± 7.1 | *66* | 45.2 ± 7.3 | *33* | 40.2 ± 5.4 |
|  | 6 months | *93* | 35.7 ± 6.8 | *62* | 37.4 ± 6.7 | *31* | 32.2 ± 5.4 |
|  | 1 year | *90* | 34.1 ± 6.9 | *61* | 35.5 ± 7.0 | *29* | 31.3 ± 5.7 |
|  | 2 years | *72* | 33.9 ± 8.1 | *45* | 36.2 ± 8.1 | *27* | 30.0 ± 6.5 |
|  | 3 years | *68* | 34.3 ± 7.3 | *43* | 36.1 ± 7.1 | *25* | 31.2 ± 6.9 |
|  | 4 years | *61* | 34.5 ± 6.8 | *39* | 35.2 ± 6.4 | *22* | 33.2 ± 7.5 |
|  | 5 years | *53* | 35.0 ± 7.6 | *34* | 35.9 ± 7.3 | *19* | 33.3 ± 8.0 |
| **TWL** (%) | Before SADI-S | *99* | - | *66* | - | *33* | - |
|  | 6 months | *93* | 17.9 ± 8.4 | *62* | 16.9 ± 7.6 | *31* | 19.7 ± 9.7 |
|  | 1 year | *90* | 21.8 ± 10.9 | *61* | 21.2 ± 10.4 | *29* | 23.3 ± 12.0 |
|  | 2 years | *72* | 23.2 ± 13.1 | *45* | 22.2 ± 11.9 | *27* | 25.0 ± 14.9 |
|  | 3 years | *68* | 21.7 ± 13.1 | *43* | 21.1 ± 12.3 | *25* | 22.9 ± 15.8 |
|  | 4 years | *61* | 19.9 ± 14.4 | *39* | 20.5 ± 12.1 | *22* | 18.9 ± 18.2 |
|  | 5 years | *53* | 19.2 ± 15.7 | *34* | 19.7 ± 13.7 | *19* | 18.3 ± 19.0 |

Data represented as mean ± standard deviation.

*CC* common channel*, BMI* body mass index*, TWL* total weight loss

**Table S2**. General patient characteristics for the total cohort and per CC length

|  | **Total cohort**  (n=99) | **CC ≤250 cm**  (n=66) | **CC>250 cm**  (n=33) | ***p* value** |
| --- | --- | --- | --- | --- |
| **Gender** (female) | 80 (80.8) | 53 (80.3) | 27 (81.8) | 0.86 |
| **Age** (years) | 43.9 ± 10.7 | 42.1 ± 9.3 | 47.6 ± 12.5 | **0.03** |
| **Weight** (kg) | 120.0 [107.0, 139.0] | 129.0 [113.8-144.0] | 108.0 [100.5-119.0] | **<0.001** |
| **BMI** (kg/m^2^) | 41.7 [38.5, 48.2] | 42.9 [39.6, 49.8] | 38.6 [36.8, 41.8] | **<0.001** |
| **Type of SADI-S**  Primary SADI-S  Secondary SADI-S | 19 (19.2)  80 (80.8) | 5 (7.6)  61 (92.4) | 14 (42.4)  19 (57.6) | **<0.001** |
| **Interval between SG and SADI-S** (months) | 35.5 [22.3, 62.0] | 36.0 [23.0, 54.5] | 34.0 [19.0, 68.0] | 0.84 |
| **Duration of SADI-S procedure** (min)^1^ | 73.5 [60.0, 94.5] | 69.0 [60.0, 91.5] | 88.0 [63.5, 100.5] | 0.058 |
| **Length of common channel** (cm) | 250 [250, 300] | 250 [250, 250] | 300 [300, 300] | **<0.001** |
| **Hospital stay** (days) | 2.0 [1.0, 2.0] | 1.5 [1.0, 2.0] | 2.0 [2.0, 3.0] | **0.02** |

Data represented as mean ± standard deviation, median [Q1, Q3] or frequency (%).

*CC* common channel*, BMI* body mass index, *SG* sleeve gastrectomy*, SADI-S* single-anastomosis

**Table S3**. Nutrient deficiencies over time before and after SADI-S, split between common channel lengths

|  | **CC ≤250 cm**  (n=66) | | | | **CC >250 cm**  (n=33) | | | | ***p* value** | | | |
| --- | --- | --- | --- | --- | --- | --- | --- | --- | --- | --- | --- | --- |
|  | *1 year* | *3 years* | *5 years* | *1 year* | | *3 years* | *5 years* | *1 year* | | *3 years* | *5 years* |  |
| **Anemia** | 17/54 (31.5) | 10/39 (25.6) | 4/26 (15.4) | 4/27 (14.8) | | 3/14 (21.4) | 1/9 (11.1) | 0.11 | | 0.99 | 0.99 |  |
| **Ferritin** | 5/49 (10.2) | 8/36 (22.2) | 4/26 (15.4) | 2/25 (8.0) | | 1/12 (8.3) | 0/8 (0.0) | 0.99 | | 0.42 | 0.55 |  |
| **Iron** | 4/21 (19.0) | 3/12 (25.0) | 0/7 (0.0) | 0/10 (0.0) | | 3/10 (30.0) | 0/7 (0.0) | 0.28 | | 0.99 | NA |  |
| **Folic acid** | 17/52 (32.7) | 7/35 (20.0) | 6/26 (23.1) | 4/23 (17.4) | | 3/12 (25.0) | 1/9 (11.1) | 0.17 | | 0.70 | 0.65 |  |
| **Vitamin A** | 0/23 (0.0) | 0/13 (0.0) | 0/4 (0.0) | 0/11 (0.0) | | 0/8 (0.0) | 0/4 (0.0) | NA | | NA | NA |  |
| **Vitamin B_1_** | 0/33 (0.0) | 0/21 (0.0) | 0/14 (0.0) | 1/19 (5.3) | | 1/10 (10.0) | 0/8 (0.0) | 0.37 | | 0.32 | NA |  |
| **Vitamin B_6_** | 0/34 (0.0) | 0/21 (0.0) | 0/15 (0.0) | 0/20 (0.0) | | 0/10 (0.0) | 0/8 (0.0) | NA | | NA | NA |  |
| **Vitamin B_12_** | 4/52 (7.7) | 5/34 (14.7) | 0/24 (0.0) | 4/26 (15.4) | | 1/12 (8.3) | 1/9 (11.1) | 0.43 | | 0.99 | 0.27 |  |
| **Vitamin D** | 18/53 (34.0) | 13/41 (31.7) | 8/28 (28.6) | 7/27 (25.9) | | 2/12 (16.7) | 1/8 (12.5) | 0.46 | | 0.47 | 0.65 |  |
| **Calcium** | 6/54 (11.1) | 5/41 (12.2) | 1/27 (3.7) | 1/27 (3.7) | | 2/12 (16.7) | 0/9 (0.0) | 0.42 | | 0.65 | 0.99 |  |
| **PTH^1^** | 28/51 (54.9) | 27/37 (73.0) | 21/26 (80.8) | 18/26 (69.2) | | 7/12 (58.3) | 5/8 (62.5) | 0.23 | | 0.47 | 0.36 |  |
| **Albumin** | 6/51 (11.8) | 3/40 (7.5) | 1/25 (4.0) | 0/18 (0.0) | | 0/12 (0.0) | 0/8 (0.0) | 0.33 | | 0.99 | 0.99 |  |
| **Zinc** | 14/21 (66.7) | 10/13 (76.9) | 3/3 (100.0) | 10/11 (90.9) | | 5/9 (55.6) | 1/4 (25.0) | 0.21 | | 0.38 | 0.14 |  |

Data are represented as valid frequency (%).

*CC* common channel*, PTH* parathyroid hormone

^1^ elevated PTH levels
