# Supplementary figures and images for: Five year outcomes of primary and secondary Single-Anastomosis Duodeno-Ileal bypass with Sleeve gastrectomy (SADI-S)
Source: Obes Surg. 2025 May 9;35(6):2160–73. doi: 10.1007/s11695-025-07888-4 (PMC12130166; doi:10.1007/s11695-025-07888-4)

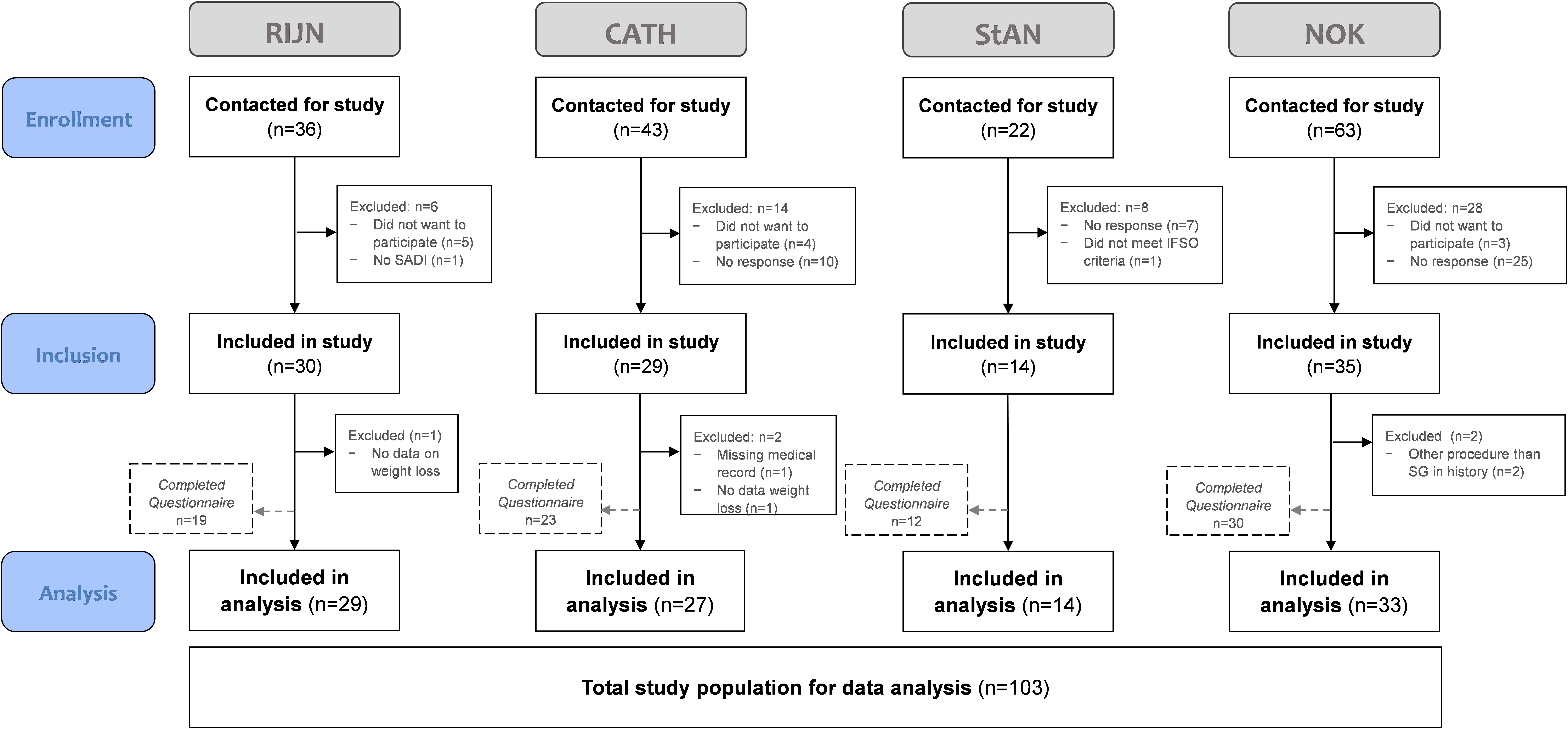

Supplement: Supplementary file 2 — Supplementary file2 (JPG 1.68 MB) [file 11695_2025_7888_MOESM2_ESM.jpg]
